# Concurrent talking in immersive virtual reality: on the dominance of visual speech cues

**DOI:** 10.1038/s41598-017-04201-x

**Published:** 2017-06-19

**Authors:** Mar Gonzalez-Franco, Antonella Maselli, Dinei Florencio, Nikolai Smolyanskiy, Zhengyou Zhang

**Affiliations:** 1 Microsoft Research, One Microsoft Way, Redmond, WA 98052 USA; 2 Event Lab, Department of Clinical Psychology and Psychobiology, University of Barcelona, Barcelona, 08035 Spain; 3 Laboratory of Neuromotor Physiology, IRCCS Santa Lucia Foundation, Via Ardeatina 306, 00179 Rome, Italy; 4 Department Electrical Engineering, University of Washington, Seattle, WA 98195 USA; 5 Nvidia Corp., Redmond, WA 98052 USA

## Abstract

Humans are good at selectively listening to specific target conversations, even in the presence of multiple concurrent speakers. In our research, we study how auditory-visual cues modulate this selective listening. We do so by using immersive Virtual Reality technologies with spatialized audio. Exposing 32 participants to an Information Masking Task with concurrent speakers, we find significantly more errors in the decision-making processes triggered by asynchronous audiovisual speech cues. More precisely, the results show that lips on the Target speaker matched to a secondary (Mask) speaker’s audio severely increase the participants’ comprehension error rates. In a control experiment (n = 20), we further explore the influences of the visual modality over auditory selective attention. The results show a dominance of visual-speech cues, which effectively turn the Mask into the Target and vice-versa. These results reveal a disruption of selective attention that is triggered by bottom-up multisensory integration. The findings are framed in the sensory perception and cognitive neuroscience theories. The VR setup is validated by replicating previous results in this literature in a supplementary experiment.

## Introduction

Humans often interact in noisy environments, where unintelligible noise or concurrent speakers masks a target speech. In such scenarios, humans are remarkably good at selectively listening to a specific target conversation while ignoring others. Research over more than five decades explored the perceptual and cognitive mechanisms that facilitate target speech segregation and recognition in noisy environments, and identified several relevant factors spanning perceptual and cognitive processing^1, 2^.

The nature of the masking and its acoustic characterization with respect to the target speech is extremely relevant^3^. Energetic masking, which might originate from both speech and non-speech sounds, shows frequency and amplitude in the same range of the target speech. This masking might hinder target-speech perception due to interference with auditory peripheral processing. On the other hand, informational masking, which consists of babbles of irrelevant intelligible speech, has stronger interference with target speech perception. This masking is likely related to stages of processing beyond auditory periphery, such as attention, perceptual grouping, short-term memory, and cognitive abilities^4^.

Indeed, informational masking depends on: the similarity of the Target’s and Mask’s voice characteristics (tone, pitch, identity, and sex); the language of the mask (native, known, or unknown); speech segmentation; target-to-mask loudness ratio; and other factors^3^.

Besides the relevant role of acoustic features, researchers have also found that selective auditory attention during active listening in noisy environments strongly relies on spatial cues^5, 6^. Thus, the lack of spatialized audio may limit understanding goal-relevant information during concurrent speaking. Many of these effects have been described in the literature as the cocktail party phenomenon^1, 2^, although the first pioneering experimental studies were targeted at improving radar operator’s communication with multiple pilots in the context of the Second World War^7, 8^.

In real-life, humans rarely rely on a single sensory modality. Auditory perception is usually strongly modulated by other afferent modalities, particularly vision^9–12^. More specifically, cognitive processes for audiovisual integration strongly characterize speech perception, in which visual cues from lip-reading affect auditory perception and vice versa^13–15^.

The impact of lip-reading on speech perception manifests in several manners. Lip-reading is well known to enhance auditory speech perception in noisy environments^16^, but it also leads to a set of cross-modal distortions in auditory perception. For example, in the ventriloquist effect, a speech sound is misperceived as dislocated towards the apparent visual source^9^. In the McGurk effect, the perception of a speech sound is shifted towards the visually articulated sound when the latter reproduces a different but related speech segment^17^. For example, hearing the syllable /ba/ when the lip movements correspond to /ga/ produces the perception of hearing/da/. Similar interactions on visual speech have been reported to not only assist phonemic restoration^18^, but also to elicit activations in the auditory cortex during silent lip-reading^19^. Strong cross-modal interference in auditory-visual speech (AVS) perception has been reported also in the opposite direction, with auditory cues affecting the perception of visual stimuli^20–22^. In a recent study, Myerson *et al*.^23^ found lip-reading to be harder during informational masking than energetic masking, providing a clear example of informational masking interference with visual speech recognition.

Another important aspect of AVS interactions, besides the multisensory-driven effects, is selective attention in environments with concurrent speakers. Selective speech perception can be enhanced by exaggerating the distance between the sources of the concurrent audio, despite a possible mislocation with the lip-reading. This was shown in a selective listening task, by presenting the video of a target-congruent visual speech in an incongruent location with respect to two competing auditory streams^24^. However, in natural environments of concurrent-speakers, competing speech stimuli are spatially coupled with their corresponding visual speech information. This might not necessarily be true for future remote telecommunications. Considering current technological advances, we can easily imagine a situation where we might not be physically present in a conversation but remotely present using immersive technologies. In this context, it is thus not clear whether and how selective attention could improve target speech perception under AVS mislocations. In our study, we explore this aspect by means of an informational masking task implemented in a highly realistic immersive setup with the support of Virtual Reality (VR) and real-time rendering of sound spatialization. Immersive setups have an additional value added when compared to physical monitors and speakers. The sense of presence, the feeling of being there, and the plausibility of the situations have been shown to deliver more realistic behaviors across social and perceptual neuroscience experiments^25, 26^.

Our experiment involved two actors speaking different sentences simultaneously. Participants performed a task from the coordinate response measure (CRM) corpus^27^, in which they selectively listen and recall a specific CALL signal from the two sentences. The CALL was randomly associated with one of the two actors. Since we recorded a new corpus (that included wide field of view stereoscopic video and audio — see supplementary material) and used a novel immersive VR setup for rendering, we first validated the setup by replicating existing literature on Information Masking Tasks with 32 participants. After we validated the setup, we manipulated the experience by modifying the AVS synchrony and congruency in presence of spatialized audio and energetic masking. While some conditions presented AVS synchrony (*SYNC*), (A*SYNC*1). In another, they matched neither the Mask nor the Target (*ASYNC*2). In a monomodal condition (*NOLIPS*), we presented only audio speech without video. We studied the different conditions in two experiments, involving 32 and 20 participants each. The results show a strong multimodal interaction when the stimuli are incongruent, offering new evidence of the level of dominance that the visual input produces over auditory speech in a mainly auditory task. Results indicate that the effects go beyond modulating the sound (i.e. phonetic restoration^18^, or McGurk^17^) and even alter selective attention.

## Results

We ran an Information Masking Task experiment on 32 participants (as detailed in the Materials and Methods section) to better understand the influences of lip-reading in the performance of a selective auditory attention task. We recorded response times and accuracy for both the ability to identify the target CALL and recall the content of the target sentence (i.e. the COMMAND). We aggregated the two metrics (response time and accuracy) into an Inverse Efficiency Score (IES), as shown in Table 1). The IES provides an informative summary of the data and compensates for possible speed-accuracy trade-offs^28^. Next, we performed a Repeated Measures analysis of variance (ANOVA) over the data. We used one within-subject factor, Condition, which was *SYNC*, *ASYNC*1 or *NOLIPS*.

**Table 1:** Performance Results. Mean and standard error of the mean for the different conditions and performance variables (Inverse Efficiency Score (IES); Response Time (RT), and % of Correct responses).

Condition	CALL IES	CALL RT	CALL %CORRECT	COMMAND IES	COMMAND RT	COMMAND %CORRECT
*SYNC*	mean = 402; SEM = 20	mean = 358; SEM = 3.9	mean = 92%; SEM = 2.5%	mean = 1051; SEM = 39	mean = 878; SEM = 7.8%	mean = 86%; SEM = 2.3%
*ASYNC*1	mean = 426; SEM = 25	mean = 361; SEM = 4	mean = 89%; SEM = 2.6%	mean = 1385; SEM = 102	mean = 890; SEM = 7.8	mean = 71%; SEM = 3.41%
*NOLIPS*	mean = 413; SEM = 26	mean = 359; SEM = 3.5	mean = 91%; SEM = 2.7%	mean = 1127; SEM = 44	mean = 882; SEM = 7.6	mean = 80%; SEM = 2.2%

In order to better understand the interactions between audio-visual modalities during selective attention, in our first experiment we compared the performance of having synchronous visual and auditory speech for both the mask and the target (*SYNC*), having only the auditory speech input (*NOLIPS*),and having the visual speech for the target matching the mask (*ASYNC*1).

**Figure 1:**
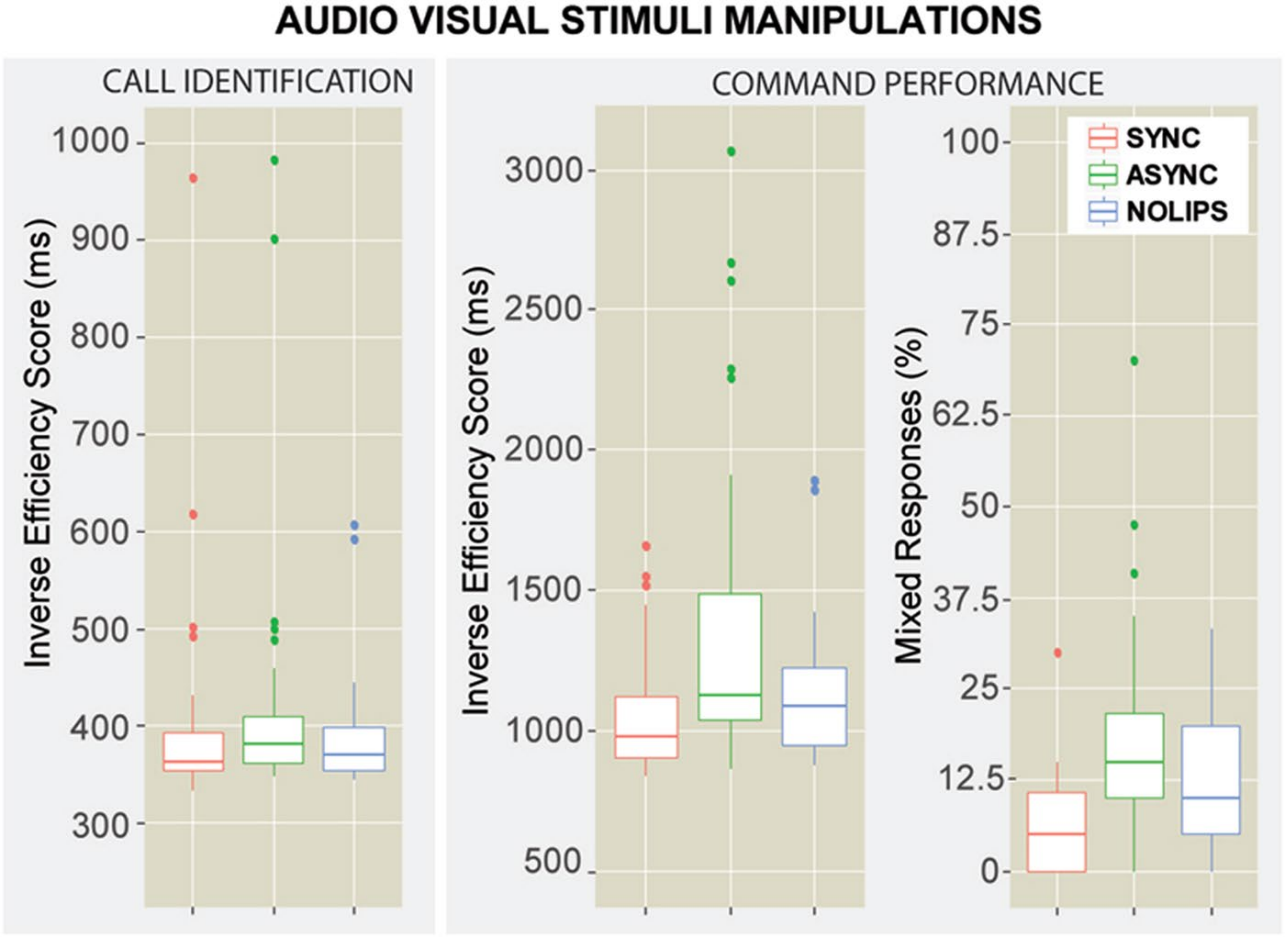
Audio Visual Stimuli (AVS) Manipulations results. Box-plots representing the Inverse Efficiency Score (IES) for the AVS conditions: *SYNC*, *ASYNC*1, *NOLIPS*. On the left the CALL identification IES, on the right the COMMAND performance and % of mixed responses.

The Repeated Measures ANOVA on the CALL identification IES revealed no significant differences across conditions (*F*(2,62) = 2.34, *p* = 0.1), as shown in Fig. 1. Since all conditions in this experiment included the same unintelligible background noise and spatialized audio and only modified lip-movements to maximize the lip-reading effects, the lack of differences in the CALL identification are likely due to dominance of auditory modality during this part of the task. Nevertheless, a deeper analysis of the unaggregated metrics showed that participants identified with higher accuracy the CALL of the target actor in the *SYNC* condition than in the *ASYNC*1 condition in a post-hoc pairwise comparison (*t* = 2.4, *p* < 0.04).

We found stronger effect on the IES for the COMMAND recall, *F*(1.22,37.82) = 11.12, *p* = 0.0009, (*ε* = 0.61), as shown in Fig. 1 and Table 1), indicating a greater influence of audiovisual speech interactions. The post-hoc pairwise comparison revealed that participants were significantly less efficient recalling the COMMAND in the *ASYNC*1 condition than in the *SYNC* and *NOLIPS* condition (*t* = −4.5, *p* = 0.001 and *t* = −3.4, *p* = 0.003, respectively). However, no differences were found between the *SYNC* and the *NOLIPS* condition (*t* = −1, *p* = 0.56). A deeper post-hoc analysis of the unaggregated metrics showed that participants made more errors in reporting the COMMAND of the target actor in the *ASYNC*1 condition than in the *SYNC* condition (*t* = 5.8, *p* < 0.0001) and in the *NOLIPS* (*t* = 3.6, *p* = 0.0015). They also showed a trend of performing better in the *SYNC* condition relative to the *NOLIPS* one (*t* = 2.1, *p* = 0.09).

Additionally, we investigated how many errors in the COMMAND recall represented mixed-up responses in which participants recalled the color or number said by the mask instead of the target. This was a good metric to evaluate the dominance of the visual modality because in the case of the ASYNC1 condition, the lips of the target corresponded to the speech of the mask. Therefore, more jumbled responses in that condition would be due to the visual manipulation retargeting auditory selective attention from the Target to the Mask. We performed a Repeated Measures ANOVA on the total mixed-up responses and found a significant effect for the conditions *F*(1.54,47.74) = 14.13, *p* < 0.001, (*ε* = 0.77) (Fig. 1). The post-hoc pairwise comparison showed that more mixed-up responses were present in the *ASYNC*1 condition (18 ± 14.4%) than in the *SYNC* condition (6 ± 7.14%, *witht* = −5.3, *p* < 0.001), and in the *NOLIPS* condition (12 ± 9.7%, *witht* = −2.7, *p* = 0.02). More jumbled responses were also found in the *NOLIPS* condition compared to the *SYNC* condition (*t* = 2.6, *p* = 0.03). These results not only show how the visual modality improved phonemic restoration when multimodal congruency was present (*SYNC*) compared to the only-auditory modality (*NOLIPS*), but also that speech visual cues were strong enough to disrupt the mainly auditory task when asynchronous (*ASYNC*1).

We further explored the correlations between the different IES performance metrics for the different AVS conditions and the responses of participants to the demographic and post-exposure questions completed in the first phase of the experiment. Lip-reading was not an explanatory factor for the performance on the CALL IES (*p* = 0.43, *ρ* = 0.10) or COMMAND IES (*p* = 0.72, *ρ* = −0.04) in the non-synchronous AVS conditions, despite participants feeling a stronger Presence Illusion and looking significantly more at the actors (*p* < 0.001, *ρ* = 0.47). In contrast to the results showing that lip-reading helped in the synchronous conditions (*p* < 0.008, *ρ* <−0.27) (see Supplementary Materials for more details on the synchronous validation). This can be explained by the fact that looking at the actors did not help with the asynchronous conditions, as the lips did not correspond to the audio. The demographics between groups show that gamers were more effective with CALL IES (*p* = 0.016, *ρ* = −0.30) and COMMAND IES (*p* = 0.002, *ρ* = −0.45), while previous Familiarity of the actors was not explanatory (*p* > 0.7).

### Control Experiment

We explored different types of asynchrony with the target’s lip motion in a control experiment to understand the lip-reading effects found in the main experiment better. In principle if the effects found in the *ASYNC*1 condition were only due to AVS interactions, an alternative asynchronous condition in which the lips were completely unrelated to the mask (*ASYNC*2) would produce similar effects. On the contrary, if the effects due to *ASYNC*2 were less intense than the *ASYNC*1, that could indicate a multimodal interaction between the mask audio and the target lip movements, specifically a retargeting of selective attention to the mask audio due to reading the target’s lips.

In a similar way to the main experiment, participants (n = 20) performed an additional Information Masking Task with three conditions (*SYNC*, *ASYNC*1, *ASYNC*2). We calculated the Inverse Efficiency Score (IES), as shown on Table 2, and ran the Repeated Measures of ANOVA with one within-subjects factor, Condition; it was set to *SYNC*, *ASYNC*1, *ASYNC*2.

**Table 2:** Performance Results Control Experiment. Mean and standard error of the mean for the different conditions and performance variables (Inverse Efficiency Score (IES); Response Time (RT), and % of Correct responses).

Condition	CALL IES	CALL RT	CALL %CORRECT	COMMAND IES	COMMAND RT	COMMAND %CORRECT
*SYNC*	mean = 364; SEM = 4.3	mean = 351; SEM = 3.2	mean = 96%; SEM = 1.1%	mean = 1255; SEM = 70	mean = 984; SEM = 9.9	mean = 81%; SEM = 3.2%
*ASYNC*1	mean = 377; SEM = 7.6	mean = 353; SEM = 2.9	mean = 93%; SEM = 1.4%	mean = 1444; SEM = 73	mean = 994; SEM = 6.6	mean = 71%; SEM = 3.3%
*ASYNC*2	mean = 377; SEM = 8.5	mean = 353; SEM = 2.9	mean = 94%; SEM = 1.8%	mean = 1413; SEM = 126	mean = 994; SEM = 8.7	mean = 77%; SEM = 4.2%

**Figure 2:**
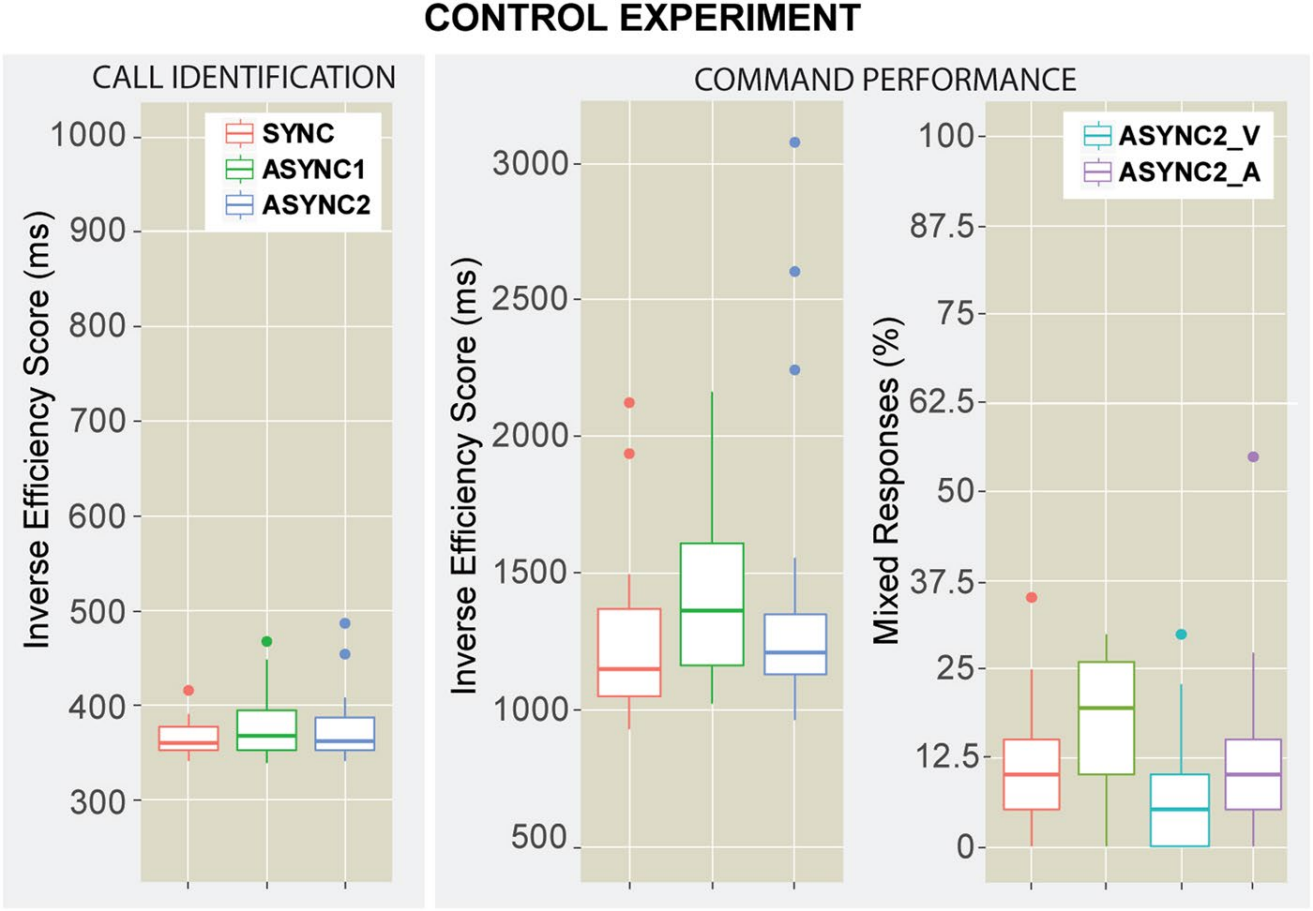
Control Experiment Results. Box-plots representing the Inverse Efficiency Score (IES) for the AVS conditions: *SYNC*, *ASYNC*1, *ASYNC*2. On the left the CALL identification IES, on the right the COMMAND performance and % of mixed responses (*SYNC*, *ASYNC*1, *ASYNC*2_*V*_, *ASYNC*2_*A*_).

We found no significant effects on the CALL identification, which is aligned with the main experiment results, showing that the identification part of the experiment relied mostly on the auditory stimuli, hence supporting the idea that lip reading was not critical for this first task. However, a significant effect was found on the IES for the COMMAND recall, *F*(1.72,32.68) = 3.62, *p* = 0.03, (*ε* = 0.86); see Fig. 2, Table 2. The post-hoc pairwise comparison revealed that participants were significantly less efficient at recalling the COMMAND in the *ASYNC*1 condition compared to the *SYNC* condition (*t* = −2.5, *p* = 0.04). When looking at the percentage of correct responses, the differences were even more significant, particularly on the COMMAND recall errors, with *F*(1.96,37.24) = 6.2, *p* = 0.004, (*ε* = 0.98), as seen in Fig. 2, Table 2. Furthermore, the post-hoc pairwise comparisons showed significantly more errors in the *ASYNC*1 than the *SYNC* condition (*t* = 3.5, *p* = 0.003). We found no significant differences with the *ASYNC*2 condition (*p* > 0.1).

To investigate how many errors were induced by the different asynchronous lip manipulations we calculated the mixed-up responses, i.e. those responses in which participants recalled the color or the number said by the mask or mouthed by the target’s lips instead of stated in the target’s audio. We calculate two mixed-up metrics: *ASYNC*2_*V*_ shows the percentage of times in which the participant responded with what the target lips were mouthing, while *ASYNC*2_*A*_ shows the percentage of times in which the participant responded with what the mask was saying. In the *ASYNC*1 condition the target lips matched the mask; hence there was only one mixed-up metric. While in the *ASYNC*2 condition, mixed-up responses would be due to either silent lip-reading of the target (*ASYNC*2_*V*_) or to the inherent errors of the mask audio (*ASYNC*2_*A*_). Further, the *ASYNC*2_*A*_ percentage serves as the baseline of mixed-up responses due only to the mask audio during non-relevant asynchronous lip motion. We performed a Repeated Measures ANOVA within-subjects with the conditions errors (*SYNC*, *ASYNC*1, *ASYNC*2_*V*_ and *ASYNC*2_*A*_) and find a significant main effect *F*(1.92,36.48) = 11.04, *p* < 0.001, (*ε* = 0.64), as seen in Fig. 2. The post-hoc pairwise comparison showed that more mixed-up responses were present in the *ASYNC*1 condition (18 ± 2.1%) than in the *SYNC* condition (11 ± 1.8%, *witht* = −3.1, *p* < 0.001), and more than in the *ASYNC*2_*V*_ condition (6 ± 1.7%, *witht* = −5.019, *p* < 0.001), and also more than in the *ASYNC*2_*A*_ condition (12 ± 2.82%, *witht* = 2.6, *p* = 0.04). The number of mixed-up responses for the *ASYNC*2 produced by lip-reading (*ASYNC*2_*V*_) and the mask audio (*ASYNC*2_*A*_) were not significantly different (*p* > 0.1).

## Discussion

Previous studies have shown that under specific circumstances the visual input on an AVS task can modulate the perception of sounds, thereby producing well-known phonemic restoration^18^ or McGurk^17^ effects. In the current study, we show how these results might have stronger ramifications affecting also selective attention and semantic interpretation beyond multimodal integration.

Interestingly, in the case of speech-on-speech informational masking, cognitive mechanisms are heavily modulated by spatial attention^5^, associated with the cocktail party phenomena^29^. Previous research even suggested that selective spatial attention modulated bottom-up informational masking of speech when only the auditory modality was presented^5^. In our study, we further investigate the relationships between the bottom-up mechanisms of multisensory integration and the top-down attentional processes. We manipulate both mechanisms using asynchronous conditions in which target’s lips and speech are mismatched. This mismatched condition disrupts bottom-up integration processing. In a variation, the target lips articulated the mask audio (*ASYNC*1), thus generating a cognitive incongruence that could induce top-down attentional modulations. Under normal circumstances, selective spatial attention would help participants to focus on the target speaker and help dissociate the masking.

Our results suggest that, when the mask audio is synchronized with the lips of the target (the *ASYNC*1 condition), visual speech cues could direct auditory selective attention to the mask speech, suggesting visual dominance in AVS integration. In this scenario, the visual speech, i.e. lip-reading, gains importance and highjacks the auditory selective attention, introducing more inefficient processing with more mistakes, mixed-up responses, and longer response times.

During the Information Masking Task audio acts as the dominant modality while the visual input acts as a modulator that can increase the confidence of the results (*SYNC* versus *NOLIPS*, or *ASYNC*2 conditions). However, in our study, by introducing a subtler AVS manipulation, we showed that visual speech cues could further affect selective attention. It is important to notice that all lip modulations in this experiment are temporally within the range of perceived alignment^30^: 64 ms in our case is below the maximum of 200 ms. Moreover, it is important to stress that in all conditions auditory stimuli from both the target and the mask were spatially congruent with the visual location of the speakers.

Given the results, we hypothesize that in our task participants started using the visual modality more intensively after the CALL identification, having identified the target actor. This aligns with priming selective attention on the target speaker, induced by the CALL target trigger, and subsequently strong multimodal effects on the COMMAND speech perception. The results show how the visual modality improved phonemic restoration when multimodal congruency was present (*SYNC*) compared to only auditory modality (*NOLIPS*), which is aligned with multisensory integration theories^9^. When the target lips were asynchronous from both the target and the mask audio (*ASYNC*2), but still presented consistent temporal and energy alignment with the target, a lower error rate was found. Indeed this condition arguably performed even better than auditory-only modality, with no lip-reading at all (*NOLIPS*). These features on their own (energy and temporal alignment) have been shown to help understanding of speech in the past during McGurk semantic experiments^31^, but also in phonetic disorder, dyslexia, and aphasia studies. There, being able to see the lips improved understanding speech semantics, despite associated sensorimotor dysfunctions^32–34^. However, those same features (energy and temporal alignment of the visual speech with the target) did not prevent the strong attentional disruption on the *ASYNC*1 condition, when the target lips articulated the mask audio. Silent lip-reading interacted early on with the audio during multisensory integration — e.g. in a similar way to phonemic restoration^18^ or silent lip-reading^19^. This made the dissociation of the target and the mask more complex and disrupted selective attention, thus affecting the integration of bottom-up mechanisms. The underlying mechanisms that trigger the disruption can be associated with an auditory retargeting, as if the lip reading on the *ASYNC*1 target effectively turned the Mask audio into the Target audio and vice versa. This shows a strong visual-speech dominance in AVS perception^35^. Interestingly, the same confusion was not present in the *ASYNC*2 condition, when the target lips did not articulate the mask speech. Therefore, it did not disrupt spatial attention, and the visual modality did not induce as many errors. These results are a major contribution since they trace a disruption on selective attention triggered by a bottom-up multisensory integration, despite the different spatial and voice features from the actors.

This study has implications to both the field of sensory perception and cognitive neuroscience. On one hand, the study shows how selective auditory attention can be hijacked by other modalities such as vision. We hypothesize that these results might extend also to other modalities of selective attention. Additionally, this study also opens new avenues to research multisensory speech perception with advanced technology capable of providing high levels of immersion, such as Virtual Reality and real-time sound spatialization. Indeed, sensory perception can be manipulated largely through these technologies, offering powerful tools to the field of experimental perceptual and cognitive neuroscience — setups that would be very hard, if not impossible, to implement in reality can be simulated inside Virtual Reality^36–39^.

## Methods

### Experimental Design

#### Conditions

We designed three conditions under which to explore the interactions between audio-visual modalities during the Information Masking Task, in which two people speak simultaneously (temporarily aligned with a standard deviation of 62 ms)^15^. However, in these conditions the AVS were not always congruent as we were interested on the influences of lip-reading to complete the task. Lip-reading has been linked to phonemic restoration which allows the auditory system to perceptually fill in missing information^18^. Phonemic restoration is more prevalent during energetic masking, therefore, in order to maximize the visual interactions, all the conditions in this section present the unintelligible ambient noise. This induced greater need to rely on the multimodal information during the task, and not only on the auditory input. All conditions featured spatialized sound; that is, the participant felt as if the voices were coming from the mouths of the actors they saw in the stereoscopic immersive VR setup. Real-time HRTF audio processing achieved the sound spatialization effect (see more details in the stimuli rendering section). The information masking between the two actors was generated through spatialized speech-on-speech masking, in which both the mask and the target were intelligible. Additionally, unintelligible/energetic masking (i.e. the ambient noise) was presented without spatialization and was combined with the actors’ voices with a Signal to Noise Ratio (SNR) of 0 dB. The VR setup aimed to simulate the closest possible scenario to a real-life auditory state with congruent and incongruent AVS, in which only the visual stimuli were manipulated through the following conditions:

- *SYNC*: The first condition featured synchronized AVS.
- *NOLIPS*: This condition presented static images by freezing the video input in the *SYNC* condition. This provided a baseline of performance on the task with single auditory modality, without visual interaction.
- *ASYNC*1: In this condition, the visual part of the speech (lip motion) was manipulated so that the lips of the target actor in the Information Masking Task matched the audio of the mask actor. We accomplished this effect by re-mixing the audio with different video outputs at the time of rendering. Therefore, this condition introduced an asynchronous lip movement for the target speech that was at the same time synchronous with the masking speech. Note that to be realistic, the alignment between the lip movements should be under the 200ms threshold proposed as a temporal constrain for auditory-visual speech integration^30^. To achieve that, the recordings were made with a visual start prompt. Data analysis on the speech start times showed a standard deviation of only 72 ms, so no additional alignment was necessary. This constraint gives clues of the level at which phonemic restoration occurs with semantic information.
  We added an additional control condition in a control experiment that also featured *SYNC* and *ASYNC*1 to explore the effects of Silent Lip Reading on the task performance:
- *ASYNC*2: In this condition, the visual part of the speech (lip motion) was manipulated so that the lips of the target actor in the Information Masking Task did not match the audio of either actor. We accomplished this effect by re-mixing the audio with different video outputs at the time of rendering. Therefore, this condition introduced an asynchronous lip movement for the target speech that was asynchronous also with the mask, but at the same time energetically similar to the original lip motion; that is it used the same number of words and temporal alignment. This condition gives additional clues about the level phonemic restoration occurs with semantic information and selective attention when is not multimodally synchronous with the auditory stimuli.

All the conditions included spatialization of the audio and energetic background noise to enforce further lip-reading effects.

#### Participants

A total of 32 subjects participated in the main experiment and supplementary validation experiment (8 *female*, *ages* 24–55 *mean* = 36.19, *sd* = 8.24). We recruited them via an email list. The experiments followed the guidelines of the Declaration of Helsinki, so participants gave written informed consent and received a lunch card as compensation for their participation. All participants had normal or corrected vision and normal hearing, and did not present dyslexia by self-admission. Later, 20 additional subjects (4 *female*, *ages* 25–53, *mean* = 34, *sd* = 7.5) were recruited for the control experiment (10 of them were participants that returned after the main experiment).

Additionally, two male actors were recruited to record the experiment corpus. The actors were 29 and 34 years old, and they were native English speakers to account for additional confounding factors^40, 41^ and were also gender-matched to make sure that the discrimination in the task was not enhanced due to frequency differences between gender voices^5^. In this experiment the actors were not professional narrators, instead they were recruited from the research unit so familiarity with the voices could be analyzed as a mixing factor in the results for the different participants. We obtained informed consent to publish the information and images of the actors in an online publication.

#### Experimental Procedure

Microsoft Research approved the experimental protocol employed in the present study, and we collected the experimental data with each participant’s approval and written informed consent after we explained the nature and possible consequences of the studies. The anonymized data collected and the recorded corpus used in the experiments are publicly available for the community and submitted as supplementary materials to this study.

At the beginning of the main experiment, participants answered a demographic questionnaire, which also included information regarding how familiar they were with the actors or what type of gamer they were:

1. *Familiarity*. How well the participants knew the actors prior to the experiment (1 I don’t know them; 2 I know them but I am unfamiliar with their voices; 3 They’ve spoken to me once or twice; 4 I talk to them on a regular basis). Among all the participants, 17 were unfamiliar with the actors’ voices (scoring 1 or 2), and 15 knew the actors (scoring 3 or 4). We considered familiarity if at least one of the actors was known.
2. *Gaming*. Which type of gamer describes you better? (0 non-gamer; 1 casual gamer; 2 core gamer; 3 hard-core gamer). Among all the participants, 13 were non-gamers, 11 casual gamers, 3 core gamers, and 5 hard-core gamers.

To account for variance between subjects, the experiment was designed so each participant underwent all of the conditions. This allowed for more reliable Repeated Measures analysis, independent of the individual performances. We presented the experiment to the participants in two parts:

The first part presented five trials of each condition in blocks. We randomized the blocks with Latin Squares to account for learning effects. At the end of each block, participants responded to the following post-exposure questions responding their level of agreement with each sentence (1 strongly disagree; 2 disagree; 3 neutral; 4 agree; 5 strongly agree):

1. *LipReading*. Looking at the person talking helped me during the task. (*Mean* = 3.48; *sd* = 1.04).
2. *Presence*. I had the feeling that I was with two real people. (*Mean* = 3.42; *sd* = 0.98).

The second part of the experiment included trials of all the conditions in completely randomized order. Participants completed 20 trials per condition. The whole protocol was sequential and lasted a total of 30 minutes. Each trial lasted 3.6 seconds, and there was an additional 1.8 seconds pause before the participants were prompted to recall the command.

For the control experiment, participants were presented with the conditions *SYNC*, *ASYNC*1 and *ASYNC*2 in an experiment that followed the same procedure of the main experiment. They completed 20 trials per condition, and we randomized all conditions from the beginning.

#### Information Masking Task

A Coordinate Response Measure (CRM)^27^ was recorded from two actors. The audio-visual recorded corpus consisted of 8 CALLs and 32 COMMANDs per actor. We combined the CALLs and COMMANDs at rendering time into full sentences that always followed the same structure: “ready *CALL* go to *COMMAND* now”. The COMMANDs consisted of one of four colors (blue, green, red or white) followed by one of eight numbers (ranging from 1 to 8). This generated a full combinatorial of 256 individual sentences when combined with one of the 8 CALLS (Arrow, Baron, Charlie, Eagle, Hopper, Laker, Ringo, and Tiger). The recorded corpus data are publicly available in the Harvard Dataverse repository^42^.

The experiment involved the concurrent playback of sentences recorded from both actors. Each trial consisted of two coordinated sentences, one from each actor. In our experiment, we asked participants to focus on the target CALL “Charlie”. At every trial, the actor who said the Target CALL was considered the Target Stimuli, while the other actor was the Mask Stimuli. One in every four trials did not include any Target CALL; we randomly selected these. Participants were asked to selectively pay attention only to the Target Stimuli, to boost the need of auditory selective attention we implemented the Information Masking Task.

During the experiment, we asked participants to point and select the person who triggered the Target CALL as fast as possible. We measured speed and accuracy on the identification of the Target Stimuli (CALL response time and CALL accuracy). The response time for the CALL did not include the “ready” word and was measured from 10 ms before the word “Charlie” started playing (that word audio lasted 350 ms), until the participant pressed the trigger on the controller. They held the controller in their dominate hand, and the system had 0.5 ms temporal resolution. After the participants selected a Target Stimuli, a two-stage questionnaire popped up on the VR, asking to select first the color and then the number. We measured how long they took to recall the COMMAND and the accuracy (COMMAND response time and COMMAND accuracy). The system judged responses correct only when both the selected color and the number matched those spoken by the Target actor. We measured the response time for the COMMAND from the moment the first questionnaire was presented until they selected both the color and the number. We used the response time and accuracy metrics to calculate the Inverse Efficiency Score (IES) in milliseconds (as described in Equation 1, which combines speed and accuracy as an aggregate cognitive metric)^43–45^. The underlying assumption for this score is that latency and proportion of errors in cognitive tasks are dependent variables and that the separate analysis tends to complicate the interpretation.

1 $$IES=\frac{RT}{1-PE}=\frac{RT}{PC}$$

where: RT is Response time; PE is Proportion of Errors; and PC is Proportion of Correct Responses.

#### Statistical Methods

To analyze how well participants performed the Information Masking task we first extracted metrics of response time, accuracy and the aggregated IES for the CALL and COMMAND parts of the task. We then ran a Repeated Measures ANOVA with one within-subjects factor, the condition. In all cases, the Greenhouse-Geisser correction was applied to the Repeated Measures ANOVA results (indicated in the text by *ε*) when the Mauchly’s test indicated the assumption of sphericity was violated (*p* < 0.05). Main factor effects are presented in the text in the form of *F*(*dfvariable*,*dferror*) = *F *− *value*, *p* = *p* − *value*, the *ε* corrections are directly applied to the degrees of freedom and p-value when necessary.

We ran pairwise post-hoc analyses on the main factor effects across the conditions. We applied Tukey p-value adjustments in all the post-hoc analyses presented in the study to account for multiple comparisons.

Additionally, we used Spearman correlations to explore the relation between the performance results and the different questionnaires. This non-parametric testing is recommended to evaluate statistical dependences with ordinal, interval, or ranked variables. The results are presented as a combination of the p-value and the rho-value, i.e. the Spearman correlation coefficient.

We presented box-plots as the preferred data distribution visualization. In the box-plots the medians are shown as the thick horizontal lines, and the boxes represent the interquartile ranges (IQR). The whiskers extend from *L* = *max*(*p*_25_ − 1.5*xIQR*, *x*_1_) to *U* = *min*(*p*_75_ + 1.5*xIQR*, *x*_*n*_), where *p*_25_ and *p*_75_ are the 25th and 75th percentiles respectively, and *x*_1_ and *x*_*n*_ are the smallest and largest data points respectively. Points outside this range are shown individually but are not excluded from the analysis.

### Apparatus

#### Stimuli Recording

**Figure 3:**
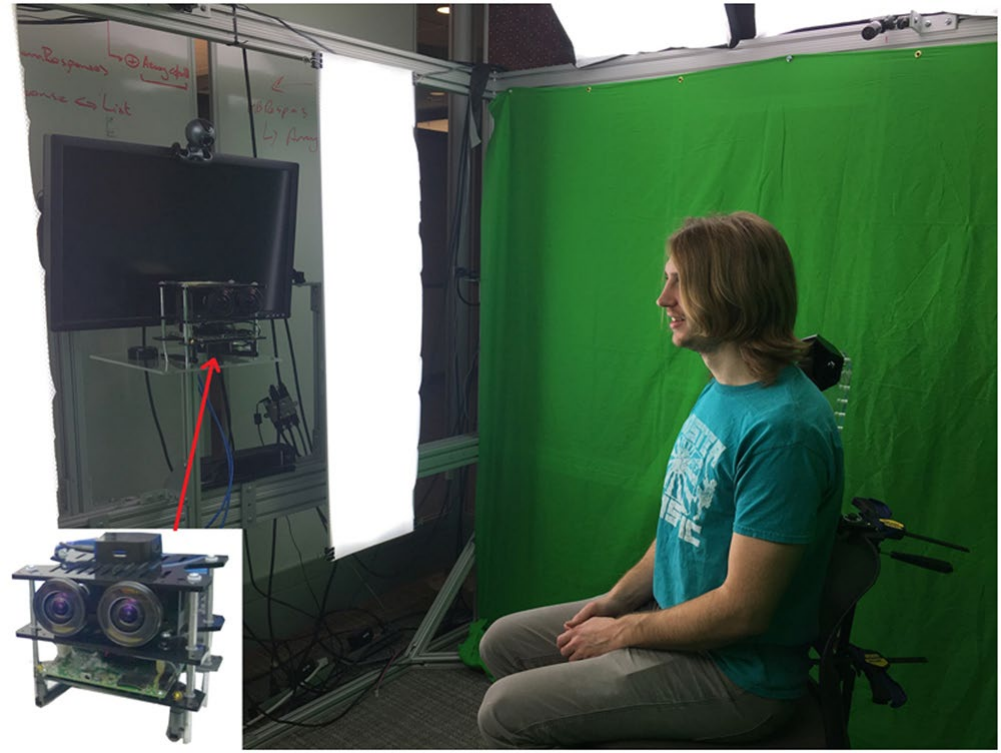
Stimuli recording setup. Recording setup with the stereoscopic camera and the actor sitting ready to record the corpus.

Our system for recording the actors consists of a custom wide-angle stereo camera system made of two Grasshopper 3 cameras with fisheye Fujinon lenses (2.7 mm focal length), reaching 185 degrees of field of view. We mounted the cameras parallel to each other, separated by 65 mm distance (the average human interpupillary distance^46^) to provide stereoscopic capturing. We encoded the video in the H.264 format at 28–30 frames per second with a resolution of 1600 × 1080 per camera/eye. The audio was recorded through a near-range microphone at a 44 kHz sampling rate and 99 kbps, and both the audio and video are synchronized to within 10 ms and stored in the mp4 format. The recording room was equipped for professional recording with monobloc LED lighting and a chromakey screen (Fig. 3). The actor sat at 1 meter from the camera recording setup and read the corpus sentences when presented on the screen behind the cameras (Fig. 3).

**Figure 4:**
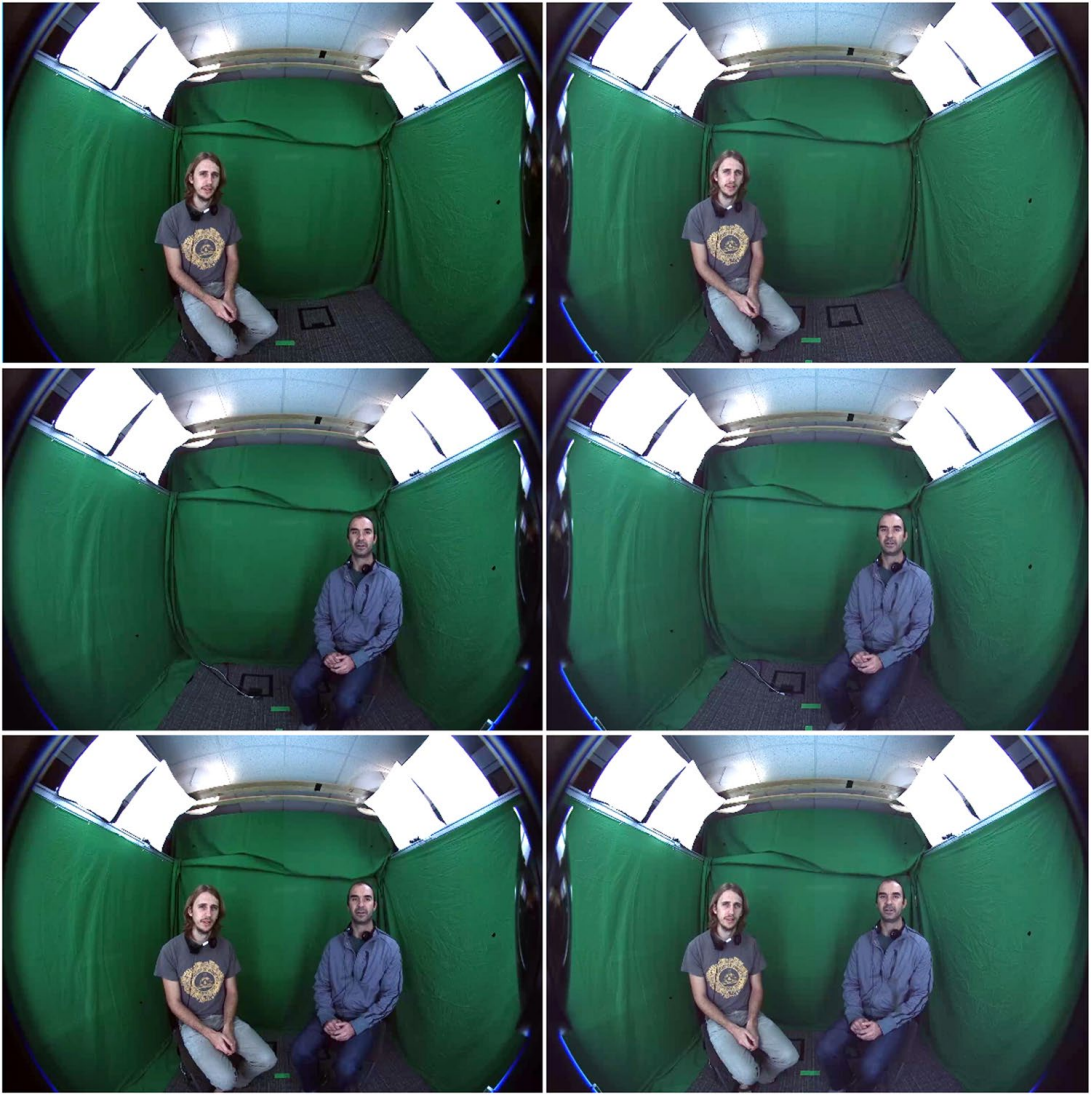
Recorded Footage. Screen shoots of the right and left-eye images as captured from the camera recording setup. The first and second rows show the two actors that were recorded separately. On the bottom the synthetically stitched scene with the two actors as the participants experienced it.

The actors were recorded separately in two sessions, seating each at 30° from the bisection, and their videos were synthetically composited at rendering time (Fig. 4). In post-processing, the audio was equalized for all words, and the video was stitched to combine the actors and generate the full the corpus. Audio was band-passed at 80 Hz to 16 kHz.

#### Stimuli Rendering

During the experiment the captured stereoscopic video and audio was rendered in Unity 3D using gstreamer1.0 for decoding. Participants experienced the video from a HTC Vive Head Mounted Display (HMD) with a 110° FoV and 2160 × 1200 combined resolution for both eyes at a 90 Hz refresh rate. The audio was played from Microsoft LifeChat LX-6000 headphones, and the actor audio sources were positioned in the 3D space so they would match the corresponding actor lips in the virtual setup. The rendering was done from an Intel Xeon E5 PC with 16 GB RAM on a GeForce GTX 980Ti graphics card. Participants used the HTC Vive Controls to perform the task, i.e. select which actor triggered the CALL and answer the questions referent to the COMMAND at the end of the trials. Both the head tracking and the controller positions and rotations were acquired using the HTC Vive system based on lighthouses that implement laser LIDAR technology with sub-millimeter precision. The correct views for left and right eyes were rendered using pre-computed UV initialized using camera intrinsic parameters calculated and using interpolation. The calibration of the fisheye equidistant camera model intrinsic and extrinsic parameters^47^ was done on 0.25 pixels re-projection error using OpenCV.

**Figure 5:**
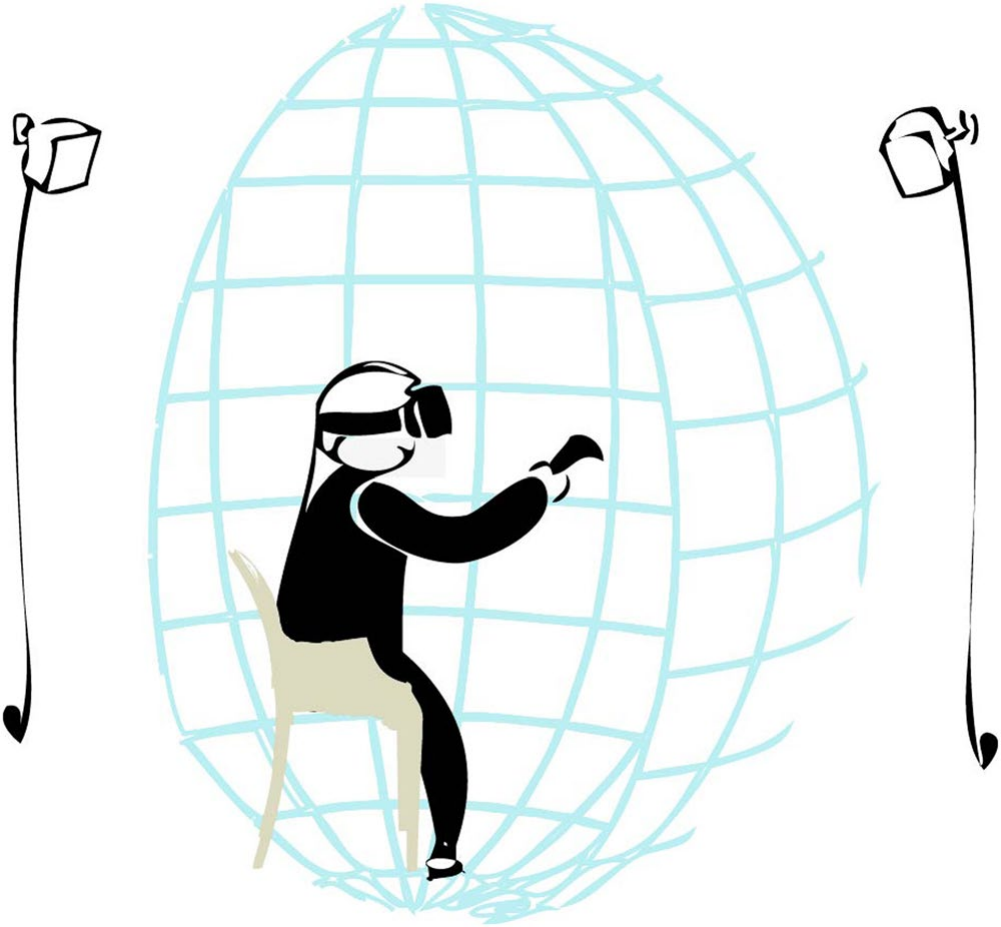
Stimuli rendering setup. Participants could look freely around the 185 degrees of stereoscopic video surrounding them through natural head movements and matched audio-visual digital panning, and performed the task using the HTC Vive controller.

Overall, the immersive stereoscopic video with a FoV of 185° combined with the head tracking provided real-time digital panning with congruent sensorimotor contingencies; that is, the head rotations and the visual input were matched. Additionally, the head tracking enabled the spatialization of the audio with respect to the participant position using a generic Head Related Transform Function (HRTF), based on the KEMAR data set^48^, which preserved the sensorimotor contingencies also for the audio motor perception. Therefore, the final rendering of the video and audio in the HMD was done in real-time based on the user’s current head pose. From the participant standpoint, this setup enabled a first- person perspective for both the video and the audio, thus generating a very immersive experience that simulated a real-life social exposure (Fig. 5).

## Electronic supplementary material

Supplementary Materials

Dataset 1
