# Supplementary material for: Concurrent talking in immersive virtual reality: on the dominance of visual speech cues
Source: Sci Rep. 2017 Jun 19;7:3817. doi: 10.1038/s41598-017-04201-x (PMC5476615; doi:10.1038/s41598-017-04201-x)
Supplement: Supplementary file 1 — Supplementary Materials [file 41598_2017_4201_MOESM1_ESM.doc]

**Supplementary Information**

Concurrent talking in immersive virtual reality: on the dominance of visual speech cues

**Authors**

Mar Gonzalez-Franco,^1,2^* Antonella Maselli,^3^ Dinei Florencio,^1^ Nikolai Smolyanskiy,^1^† Zhengyou Zhang^1,4^

**Affiliations**

^1^ Microsoft Research, One Microsoft Way, Redmond, WA 98052, USA

^2^ Event Lab, Department of Clinical Psychology and Psychobiology, University of Barcelona, 08035 Barcelona, Spain

^3^ Laboratory of Neuromotor Physiology, IRCCS Santa Lucia Foundation, Via Ardeatina 306, 00179 Rome, Italy

^4^ Dept. Electrical Engineering, University of Washington, WA 98195 Seattle, USA

* Corresponding author. E-mail: [margon@microsoft.com](mailto:margon@microsoft.com)

† Current affiliation: Nvidia Corp., Redmond, WA 98052, USA

**Supplementary Information**

**Experimental Setup Validation**

Several studies have presented Virtual Reality (VR) as a more advanced technological alternative to test body perception and multisensory integration beyond what would be possible in reality (*1*–4). With that in mind, we prepare a technological setup able of providing a fully immersive experience that simulates a real-life environment. Inside VR, participants complete an Information Masking Task with different Audio Visual Speech (AVS) conditions. To validate the experimental setup and the Virtual Reality technology for the study of AVS we ran three additional conditions to replicate important literature findings. The three conditions feature always synchronous AVS with either MONO or SPATIAL sound with either added ambient NOISE or no noise. (All the conditions and analysis are explained in the Material and Methods section of the main text).

***Conditions***

We designed three additional conditions to validate the technological setup used in the experiment. These aimed at replicating important literature findings on selective auditory attention with congruent AVS and spatialized audio during Information Masking Tasks. The results of these conditions are the Virtual Reality setup validation (see Supplementary Material):

- *SPATIAL:* The first condition featured spatialized sound with synchronized AVS; i.e. the participant felt as if the voices were coming from the mouths of the people they saw in the Virtual Reality setup. We created this effect through a real-time HRTF audio processing able of spatializing sound. We did the information masking in this condition with speech-on-speech masking, during which both the mask and the target were intelligible.
- *MONO:* A control condition, in which AVS were the same as in the *SPATIAL* condition but presented non-spatial sound; i.e. the speech of the actors was not processed through an HRTF therefore coming to both ears of the participant without spatialization.
- *NOISE:* We designed a third condition to introduce ambient noise into the *SPATIAL* condition. We intended this condition to introduce unintelligible masking rather than only intelligible masking. In the *NOISE* condition the intelligible masking was spatialized but the unintelligible masking (i.e. the ambient noise) was presented without spatialization in the same way as the *MONO* condition. This condition is the same as the *SYNC* condition previously described in the main experiment.

***Results***

We first analyze the performance on the CALL, i.e. the target actor identification (Figure S1). A significant main effect on the Inverse Efficiency Score (IES) to identify the target was found among the conditions (SPATIAL, MONO and NOISE) F(1.06,32.86)=13.69, p=0.0006, (ε = .53). The post-hoc pairwise comparison showed that participants were significantly more inefficient to identify the target actor in the MONO condition (523±271ms) than in the SPATIAL condition (398±90ms) even when NOISE was added (402±117ms, with t=-4.4, p<0.0001). No differences were found between the SPATIAL condition with and without NOISE (t=-0.13, p=0.98).

Then we analyze how well participants recalled the COMMAND (Figure S1). A significant main effect on the IES performance was found F(1.06,32.86)=34.23, p<0.0001, (ε = .53). The post-hoc pairwise comparison showed that participants performed significantly better in the SPATIAL condition (963±134ms) than in the MONO condition (1651±712ms) even when NOISE was added (1051±220ms, with t=-6.6, p<0.001). The Inefficiency Score was not significantly higher with NOISE than without noise in the SPATIAL condition (t=-0.9, p=0.59). However, when analyzing the accuracy on its own, the addition of background NOISE (86±13.2% correct) introduced significantly more errors than the original SPATIAL condition (92±9.8% correct, with t=2.3, p=0.05). The rest of the response times and accuracies for the CALL and COMMAND remained consistent with the IES results when analyzed without aggregation.

***Explanatory factors***

We explore the correlations between the different IES performance metrics for the three conditions (SPATIAL, MONO and NOISE) and demographic and post-exposure responses (Table S1).

As shown by the correlations matrix, participants who were Familiar the actors’ voices were not significantly better in any of the performance metrics. Although this might seem counterintuitive, it can be explained by the different between-subject variances in performance, which are independent of how Familiar participants were with the voices. A significant negative correlation was found between Gaming and CALL IES showing that gamers were able to identify the actor more efficiently than non-gamers.

Additionally, significant correlations were found between the Lip Reading responses and COMMAND IES and CALL IES. I.e. participants who said they looked at the person talking were more efficient in their command response and in the target identification (Figure S1). This can be explained by the higher confidence on the decisions that is generated by multisensory integration between the auditory and the visual afferent inputs, i.e. matching the audio to the lip reading improved the Phonemic Restoration (5). This effect validates our experimental setup for the study of the AVS multimodal interactions during Information Masking.

The Presence question was an important explanatory factor of the performance metrics, showing better CALL and COMMAND efficiency when participants experienced higher Presence (Figure S1). These significant correlations could be explained by the more realistic behavior: participants who find the experience more realistic achieve better performances. A significant correlation was also found between the Lip Reading and Presence factors. Indicating that people who found the whole experience more realistic also tended to look more at the actors talking. We hypothesize that this behavior derives from real social interactions in which people would look to the interlocutors while they talk, therefore it is not surprising that participants who found the experience more plausible also looked at the actors more directly.

References and Notes

1. M. González-Franco, T. C. Peck, A. Rodríguez-Fornells, M. Slater, A threat to a virtual hand elicits motor cortex activation. *Exp. brain Res.* **232**, 875–87 (2014).

2. D. Banakou, M. Slater, Body ownership causes illusory self-attribution of speaking and influences subsequent real speaking. *Proc. Natl. Acad. Sci.* **111** (2014), doi:10.1073/pnas.1414936111.

3. G. Padrao, M. Gonzalez-Franco, M. V Sanchez-Vives, M. Slater, A. Rodriguez-Fornells, Violating body movement semantics: Neural signatures of self-generated and external-generated errors. *Neuroimage*. **124 PA**, 174–156 (2016).

4. A. Maselli, K. Kilteni, J. López-Moliner, M. Slater, The sense of body ownership relaxes temporal constraints for multisensory integration. *Sci. Rep.* **6** (2016).

5. R. M. Warren, Perceptual restoration of missing speech sounds. *Science (80-. ).* **167**, 392–393 (1970).


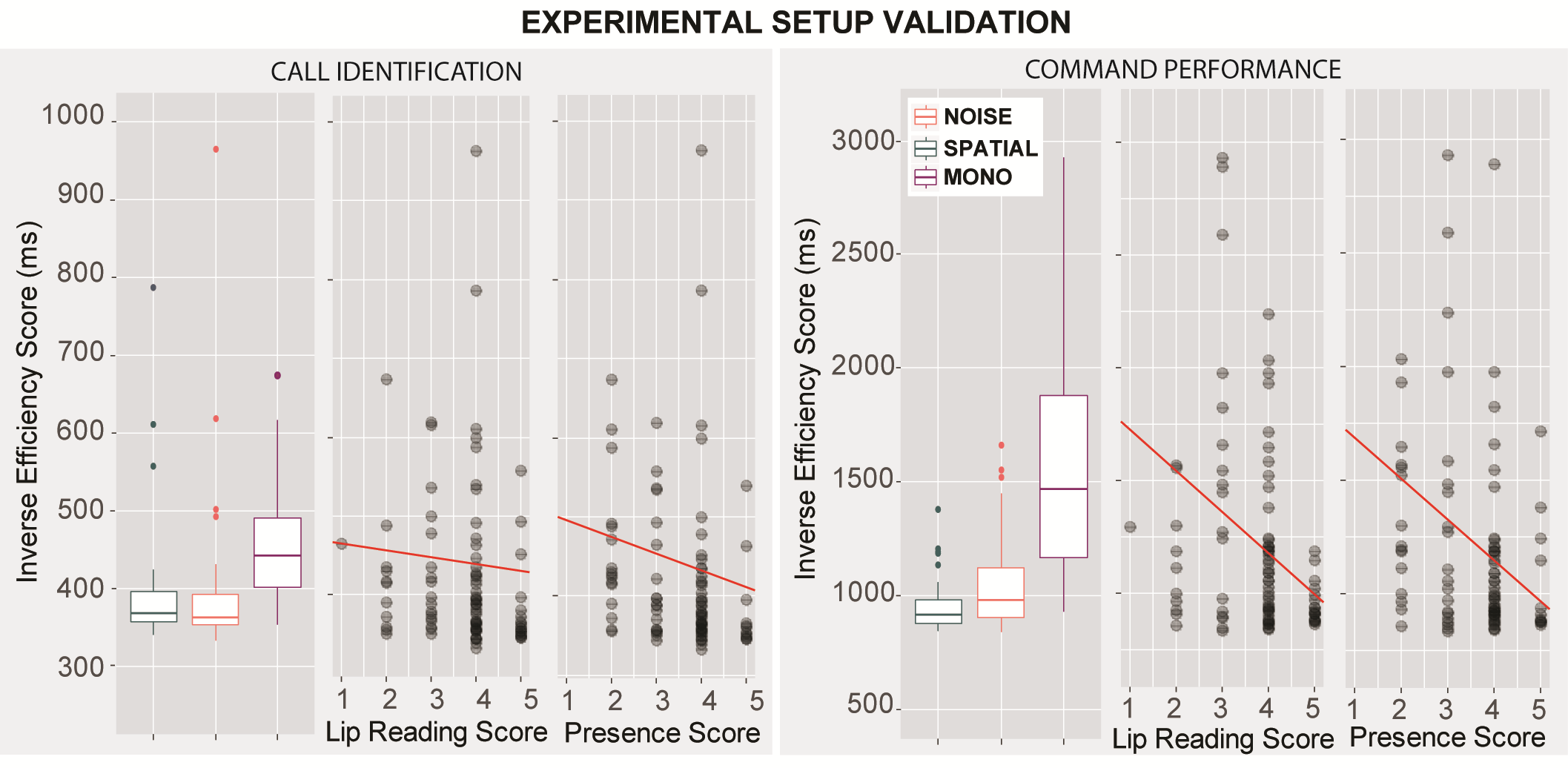


**Fig. S1.** **Experimental setup validation results.** On the left the call identification Inverse Efficiency Score (IES) for the AVS synchronous conditions (Spatial, Noise and Mono). And the correlations between the IES and the lip reading and presence scores. On the right the command performance for the same conditions and the corresponding correlations.

|  | CALL IES | COMMAND  IES | Gaming | Lipreading | Presence |
| --- | --- | --- | --- | --- | --- |
| Knows | p=0.6205 rho=0.05 | p=0.7963  rho=0.03 | p=0.7678  rho=-0.03 | p=0. 2612  rho=0.12 | p=0.1341 rho= -0.15 |
| Gaming | **p=0.0105 * rho=-0.26** | p=0.0568 rho=-0.20 |  | p=0.0899  rho=0.17 | p=0.3112 rho=0.10 |
| Lip Reading | **p=0.0081 ***  rho=-0.27** | **p=0.0012***  rho=-0.33** |  |  | **p=0.0000 ***  rho=0.42** |
| Presence | **p=0.0008 **  rho=-0.34** | **p=0.0021 **  rho=-0.31** |  |  |  |

*p<0.05, **p<0.01, ***p<0.001

**Table S1.** **Spearman Correlations and Explanatory Factors.** Correlations between the performance metrics (Inverse Efficiency Score, IES) and the different demographic and post-exposure responses for the conditions with congruent AVS (Spatial, Noise and Mono).

**Data S1.** **Corpus.** The recorded corpus used for this study is available in the Harvard Dataverse:

Gonzalez-Franco, M, 2017, "Corpus Data for: "Hearing lips: on the dominance of vision in immersive cocktail party phenomena"", doi:10.7910/DVN/KHXBBB, Harvard Dataverse, V1 <http://dx.doi.org/10.7910/DVN/KHXBBB>

**Data S2.** **Collected data.** The collected data for all the experiments and conditions.
